# Gamma Oscillations in Alzheimer’s Disease and Their Potential Therapeutic Role

**DOI:** 10.3389/fnsys.2021.782399

**Published:** 2021-12-13

**Authors:** Artemis Traikapi, Nikos Konstantinou

**Affiliations:** Department of Rehabilitation Sciences, Faculty of Health Sciences, Cyprus University of Technology, Limassol, Cyprus

**Keywords:** Alzheimer’s disease, gamma brain oscillations, sensory stimuli, auditory and visual stimulation, 40 Hz brain stimulation

## Abstract

Despite decades of research, Alzheimer’s Disease (AD) remains a lethal neurodegenerative disorder for which there are no effective treatments. This review examines the latest evidence of a novel and newly introduced perspective, which focuses on the restoration of gamma oscillations and investigates their potential role in the treatment of AD. Gamma brain activity (∼25–100 Hz) has been well-known for its role in cognitive function, including memory, and it is fundamental for healthy brain activity and intra-brain communication. Aberrant gamma oscillations have been observed in both mice AD models and human AD patients. A recent line of work demonstrated that gamma entrainment, through auditory and visual sensory stimulation, can effectively attenuate AD pathology and improve cognitive function in mice models of the disease. The first evidence from AD patients indicate that gamma entrainment therapy can reduce loss of functional connectivity and brain atrophy, improve cognitive function, and ameliorate several pathological markers of the disease. Even though research is still in its infancy, evidence suggests that gamma-based therapy may have a disease-modifying effect and has signified a new and promising era in AD research.

## Introduction

Alzheimer’s Disease (AD) is a slowly progressing, lethal neurodegenerative disorder and the most prevalent form of dementia accounting for 60–80% of the total dementia cases worldwide (Alzheimer’s Association, 2020). The core pathological hallmarks and the most dominant hypotheses of AD are the extracellular plaque accumulation formed by amyloid beta (Aβ), and intracellular neurofibrillary tangles composed of phosphorylated tau protein. Both pathologies cause a broad variety of neurotoxic cascades and massive loss of neurons and synapses in the cerebral cortex and hippocampus (Bloom, 2014).

Even though scientific knowledge has increased enormously and our understanding of the pathological mechanisms involved in AD have been well-identified, clinical trials that have targeted these mechanisms have not succeeded in identifying effective methods to treat or reverse the disease (Fan et al., 2020). Considering the alarming rise and the tremendous personal, social, and financial burden of AD, scientists have tried to move away from the well-recognized hypotheses. AD pathogenesis has recently been explored from different perspectives, such as infection, cerebral vasoconstriction and prion transmission (Jeong et al., 2018; Seminara et al., 2018; Tian et al., 2019), offering new insights into the potential treatments of AD.

A recent pioneering approach targets brain waves to treat AD pathology. Brain oscillations, particular gamma oscillations, are altered in AD patients and animal models of the disease (e.g., Jelles et al., 2008; Klein et al., 2016). On that basis, a novel approach investigates the effectiveness of a therapy based on modulation of neuronal gamma oscillations using sensory inputs, and its potential therapeutic role, signifying a new and promising era in AD research. In this review, we summarize the status of research on the potential therapeutic effect of gamma entrainment in mice models of AD and human patients.

## Aberrant Gamma Neural Activity in Alzheimer’s Disease: Evidence From Animal Studies

Gamma waves are the fastest brainwaves in the human brain, produced by synchronized electrical pulses from masses of neurons communicating with each other, resonating between approximately 25 and 100 Hz (Hughes, 2008). Several human studies have investigated their role in object representation and visual feature binding (Tallon-Baudry and Bertrand, 1999) as well as in higher cognitive functions (van Vugt et al., 2010; Miller et al., 2018; Griffiths et al., 2019). Gamma rhythms have been recorded ubiquitously across the human brain. They are being widely encountered in the visual system (Murty et al., 2018; Zhigalov et al., 2021) in addition to different cortical and subcortical brain structures (e.g., Cohen et al., 2009; van der Werf et al., 2010; Manabe and Mori, 2013). What is more, it has been suggested that gamma-band synchronization supports fundamental functions critical for various cognitive processes (Bosman et al., 2014). All this evidence reveals gamma oscillations’ multifunctionality, which supports a wide range of both sensory and higher cognitive functions (for further information on gamma oscillations, please see Bosman et al., 2014; Cannon et al., 2014; Fries, 2015; Cole and Voytek, 2017).

Mounting evidence suggests that gamma rhythms are also prominent in the hippocampus, playing a crucial role in memory functions (e.g., Colgin and Moser, 2010; Carr et al., 2012; Buzsáki, 2015). It is, therefore, not surprising, that disorders characterized by memory impairment, such as AD, are associated with aberrant activity of gamma oscillations (Mably and Colgin, 2018). The presence of gamma oscillations has been identified *in vivo* in the hippocampus and the entorhinal cortex of healthy rodents (Penttonen et al., 1998), while changes in this brain activity have been evident in several rodent models of AD. In fact, Klein et al. (2016) reported changes in rodents’ gamma oscillations in the entorhinal cortex at an early stage of the disease. Another study found impaired theta-gamma cross-frequency coupling in the hippocampus before plaque formation, in 1-month old transgenic mice (Goutagny et al., 2013). Verret et al. (2012) identified that both AD patients and transgenic mice presented dysfunctions on the parvalbumin cells and inhibitory synaptic activity which resulted in aberrant gamma oscillatory activity and cognitive disfunction. Restoring the levels of the interneuron-specific and parvalbumin cells sodium channel proteins had profound and beneficial effects on gamma brain activity and cognitive function. Goutagny et al. (2013) indicated that the reduction of slow gamma activity (25–50 Hz) in the hippocampal area CA1 can result in impaired memory function. Similar findings were reported by Mably et al. (2017) who demonstrated that decreased gamma activity in mice models of AD can result in memory dysfunction. Cumulatively, current evidence suggests that gamma brain activity is decreased in mice models of the disease, causing cognitive dysfunction. The exact mechanisms responsible for this alteration are not known, however, it has been suggested that synaptic and neuronal functions are affected by either soluble Aβ or its fibrillary forms (Walsh and Selkoe, 2004), contributing to network alterations (Palop and Mucke, 2016). In rodents, these changes are reported to appear before plaques formation (Iaccarino et al., 2016; Etter et al., 2019).

Evidence that aberrant gamma activity and cognitive dysfunction arise in mice models of AD before the accumulation of amyloid plaques provides convincing evidence that abnormalities in gamma brain oscillations may represent an early biomarker for AD (Goutagny et al., 2013; Mably and Colgin, 2018). Nevertheless, the question of whether the aberrant gamma oscillations activity is responsible for the observed cognitive impairment in AD patients or if it is just another by-product of the disease pathology, which produces the cognitive symptoms, remains to be answered. Additionally, it is unclear whether inducing gamma oscillations in transgenic mice can affect AD pathophysiology and cognition. It has been shown, however, that steady-state brain gamma oscillations can be stimulated or driven by sensory stimulation (e.g., Ross et al., 2013) and that neuronal activity has the potential to modulate the Aβ load (Cirrito et al., 2005; Bero et al., 2011).

## The Effects of Gamma Stimulation in Mice

In light of accumulating evidence, recent studies demonstrated that restoration of gamma oscillations has the potential to alleviate AD pathology and cognitive function in mice. Iaccarino et al. (2016) used a non-invasive brain stimulation technique to manipulate gamma brainwaves. Specifically, the authors used light flicker stimulation at three different frequencies (20, 40, 80 Hz), constant light or dark, and random flicker to treat the pathology of 5XFAD mice. After only 1 h of stimulation the authors observed reduction of Aβ levels in visual cortex by almost 60%, compared with dark controls. Another critical finding was the transformation of microglial morphology consistent with increased phagocytic activity and the elevation of microglia/Aβ interactions. These effects were specific to the 40 Hz light exposure as neither constant light nor 20 Hz, 80 Hz or random flicker led to significant changes in mice pathology. After treating 5XFAD mice for 1 h daily over 7 days, the investigators found that 40 Hz visual stimulation significantly decreased both soluble and insoluble Aβ_1–40_ and Aβ_1–42_ levels and reduced the accumulated amyloid pathology in visual cortex by almost 67%, compared with dark controls. Finally, they observed reduction of the tau tangles in a mouse model of tauopathy, indicating the generalization of 40 Hz stimulation effect to other pathogenic proteins. Interestingly, these findings were replicated in several mice models, including WT, 5XFAD, and APP/PSI (i.e., mice genetically engineered to exhibit specific AD pathologies; for reviews see Elder et al., 2010; Oblak et al., 2021), suggesting that the effects are not specific to one animal model.

In a more recent study Martorell et al. (2019) presented mice models of AD with noise stimuli at 8 Hz, 40 Hz, 80 Hz and random stimulation tones for 1 h, daily, for 7 days. The authors reported that only 40 Hz stimulation produced similar with Iaccarino et al. (2016) results, that extended from the primary auditory cortex to the hippocampus and were accompanied by memory improvements. When the authors combined the auditory with the visual (A + V) 40 Hz stimulation they observed reduction of amyloid dispositions not only to the primary sensory cortices and the hippocampus, but also to the medial prefrontal cortex and within the whole neocortex. Amyloid reduction was frequency-specific, as it was observed only at 40 Hz but not at 8 Hz, 80 Hz or at random A + V frequency stimulation. Additionally, the authors observed profound glial responses after the concurrent A + V 40 Hz stimulation to the aforementioned areas, vs. visual or auditory stimulation alone and no stimulation controls. Finally, from a study of the same research group (Adaikkan et al., 2019), it became evident that 40 Hz visual stimulation entrains gamma oscillatory activity not only to the visual cortex but also to the hippocampus, the prefrontal and the somatosensory cortices. In addition, gamma activity was not enhanced only at local networks but also between these areas, indicating that 40 Hz light stimulation improves inter-area communication, whereas 80 Hz stimulation did not produce similar results. Finally, the authors delivered 40 Hz visual stimulation or no stimulation to tau P301S mice for 22 days. It was found that prolonged daily 40 Hz visual stimulation had a neuroprotective effect, in terms of reduction of neuronal and synaptic loss in broad brain areas, which was accompanied by cognitive improvements.

Overall, these studies provided evidence that inducing gamma oscillations through sensory 40 Hz stimulation has the potential to ameliorate several of AD’s key neuropathologies, such as amyloid plaques, neurofibrillary tangles, and neuronal and synaptic loss, in several mice models of AD. While the exact mechanisms under which the observed changes occurred remain to be determined, these findings raised the interesting question of whether an increase in gamma frequency neural activity in humans can be a promising strategy to alleviate AD’s pathological changes and hence, to restore patients’ cognitive deficits. In any case, generalization of these 40 Hz protocols has gained interest and it is currently a promising avenue (McDermott et al., 2018).

In view of the above findings, Yao et al. (2020) used light stimulation to investigate its effect on circadian rhythm in AD mice models. The authors used APP/PSI mice models of AD and their non-transgenic wild-type (WT) littermates and randomly divided them into no-stimulation or 40 Hz light therapy groups. They found that 40 Hz light flicker for 1 h, daily, for a 30 day period, ameliorated circadian rhythm disorder compared to the no-stimulation condition, which is suggested to represent a primary component of the causal pathway, which leads to AD pathogenesis and accelerates its progression (Vanderheyden et al., 2018). Furthermore, the authors observed induced gamma activity in visual cortex and reduction of Aβ and tau production in the hypothalamus of the APP/PSI mice. Additionally, they reported an increase of key clock proteins expression (e.g., BMAL1, CLOCK, and PER2), and a restoration of the hypothalamus electrophysiological changes. Importantly, the 30-days light therapy proved safe, leading to no adverse side effects to mice’s physiological status. These effects were evident only in the APP/PSI mice that received the 40 Hz light treatment but no such effects were found in the no-stimulation condition. Taken together, this evidence suggests that 40 Hz sensory stimulation may also have the potential to treat the circadian rhythm disorder presented in AD (Homolak et al., 2018; Phan and Malkani, 2019; Pak et al., 2020).

Bobola et al. (2020) examined whether it is possible to safely alleviate AD pathophysiology in mice models, and hence to replicate the work of Iaccarino et al. (2016), using transcranial focused ultrasound pulsed at 40 Hz. The authors used 40 Hz ultrasound without contrast agents and observed microglia activation that co-localized with amyloid plaques after just 1 h of application, as compared to sham stimulation on age-matched mice. After 5 days of transcranial ultrasound therapy, the mice’s Aβ burden reduced by almost 50% in the ultrasound exposed brain and by 28% in the CA1 area, in contrast to sham therapy. These results compare closely with those observed by medications applied to mice models for substantially prolonged time periods (Bhattacharya et al., 2014; Chandra and Pahan, 2019). Overall, even though the application of 40 Hz transcranial ultrasound did not lead to the generalization of the effects throughout the brain as with the combination of auditory and visual sensory stimulation by Martorell et al. (2019), the results demonstrated that it is a safe and effective intervention and further research is needed in this area.

The aforementioned findings provide compelling evidence on the positive effects of audiovisual sensory gamma stimulation on AD pathology in mice. Additionally, it should be noted that Zhen et al. (2017) demonstrated that neuropathological changes and cognitive function can also be alleviated through gamma burst magnetic stimulation, delivered *via* deep-brain reachable low field magnetic stimulation (i.e., a non-invasive technique designed to intervene with the brain’s neuronal activity), in mice models of the disease. Following the robust animal data, research is now focusing on investigating whether these findings would translate into human AD patients.

## Sensory Gamma Frequency Stimulation in Alzheimer’s Disease Patients

The accumulating evidence on gamma entrainment through visual and auditory stimulation in mice was a major stride toward establishing the importance of gamma brain waves, particularly at 40 Hz, in the amelioration of AD neuropathology. The research on the therapeutic efficacy of gamma sensory stimulation in AD patients, although still in its infancy, provides encouraging data. Clements-Cortes et al. (2016) evaluated the effects of 40 Hz sound stimulation vs. non-rhythmic visual stimulation in AD patients. The study indicated that somatosensory stimulation, *via* a device (i.e., NextWave chair) which produced 40 Hz sound waves through six speakers, had a significant effect on the cognitive function of patients with mild to moderate AD, in contrast with the visual stimulation which involved nature pictures displayed on a television screen. While patients’ gamma brain activity or possible changes on AD pathology were not assessed, this study provided solid evidence regarding the efficacy of gamma-based treatment in AD. More recently, Suk et al. (2020) developed a light and noise delivery device and stimulated at 40 Hz cognitively healthy, AD, and epileptic patients while their electrophysiological responses were recorded. The concurrent audiovisual stimulation was proved safe in all subject groups, including the epileptic patients, while induced highly coordinated 40 Hz oscillations. Even though patients’ cognitive abilities were not examined, this study provided preliminary evidence regarding the safety and feasibility of 40 Hz sensory stimulation in AD patients.

To investigate the efficacy of sensory stimulation as a novel disease modifying therapy in AD patients, Chan et al. (2021) conducted a longitudinal, placebo-controlled trial. Specifically, 15 patients with mild AD were recruited and divided into experimental and control groups. Both groups were given the same sensory stimulation device to use at home for 1 h daily. The devise was programmed to deliver constant white light and noise to the control group and synchronized audiovisual stimulation at 40 Hz to the active group. AD patients who received real auditory and visual stimulation treatment daily, over a period of 3 months, presented reduced loss of functional connectivity, improved memory performance and ameliorated sleep markers in relation to controls. Additionally, the active group showed less brain atrophy and more importantly did not show further hippocampal atrophy or ventricular expansion in relation to the control group, indicating a possible delay in the disease progression. In the same manner, He et al. (2021) recruited 10 patients with prodromal AD who received daily 40 Hz audiovisual sensory stimulation for 1 h over the course of 4 or 8 weeks. Even though, control group or sham stimuli were not included for comparison reasons, the study indicated that the treatment was safe and resulted in improved functional connectivity in the default mode network and altered cytokines and immune factors in the cerebrospinal fluid. Overall, both studies provided compelling evidence that gamma-based therapy, through visual and auditory stimulation, may have a disease modifying effect and may be used to alleviate AD pathology. Table 1 summarizes the clinical studies that applied gamma frequency brain stimulation in AD patients.

**TABLE 1:** Summary of clinical studies that applied gamma frequency brain stimulation to Alzheimer’s Disease (AD) patients.

References	Stimulus type (frequency)	Method of stimulation	N	Control condition	Outcome measures	Results
Clements-Cortes et al., 2016	Sound; vibrotactile-somatosensory (39.96–40.06 Hz)	6 × 30’ sessions of sound administered through the six speakers of the NextWave chair	18 from mild to moderate AD patients	Visual stimulation (nature images displayed on TV)	SLUMS, OERS, and behavioral observation	Statistically significant improvement on SLUMS score by 0.5 units for each session; strongest impact on mild and moderate AD patients
Suk et al., 2020	Concurrent light and sound	1 session of 1 h through a device that delivered light and sound	17 AD patients	No control stimuli	EEG, iEEG	Widespread and coordinate 40 Hz oscillations in comparison to light or sound stimulation alone; safe and feasible potential therapy for AD
Kehler et al., 2020	tACS (40 Hz)	2, 30-min tACS sessions/day + cognitive exercises, 5 days/week for 4 weeks	17 MCI or mild to moderate AD patients	Cognitive exercises with no-tACS	WMS-IV and MADRS	Memory improvement in the active and sham group which maintained significantly better in the active group after 1 month
He et al., 2021	Light and sound (40 Hz)	4 or 8 weeks of 1 h daily sensory device-based stimulation	10 MCI due to AD patients	No control stimuli	MRI, EEG, venous blood draws, lumbar punctures, and cognitive testing	Increase functional connectivity in the DMN and alter cytokines and immune factors in the CSF after 8 weeks of therapy. Sensory gamma stimulation is safe, tolerable, and feasible
Chan et al., 2021	Light and sound (40 Hz)	3 months of 1 h daily concurrent stimulation through a non-invasive sensory stimulation device	15 mild AD patients	Constant light and white noise	EEG, MRI, fMRI, actigraphy recordings, cognitive assessments	Induce gamma entrainment; reduce ventricular dilation; preservation of hippocampal volume; improve functional connectivity in the DMN; cognitive and circadian rhythmicity improvement. Safe and feasible therapy
Benussi et al., 2021	tACS (40 Hz)	1 session of 1 h tACS over Pz	20 MCI due to AD patients	Sham stimulation	Cognitive assessments and indirect measures of cholinergic transmission evaluated using TMS	Statistically significant improvement in memory performance and restoration of intracortical connectivity measures of cholinergic neurotransmission in comparison to sham therapy
Liu et al., 2021	TMS (40 Hz)	12 sessions/2,400 pulses each, over the course on 4 weeks over the bilateral angular gyrus (1,200 pulses each)	37 AD patients and 41 healthy controls	Sham stimulation	Neuropsychological assessments, MRI, EEG	Cognitive function improvement, gamma-band power enhancement of the left temporoparietal cortex, local, long-distance, and dynamic connectivity increasement within the brain, promotion of more strong information flow

*tACS, transcranial alternating current stimulation; TMS, transcranial magnetic stimulation; MCI, mild cognitive impairment; TV, television; SLUMS, St. Louis university mental status test; OERS, observed emotion rating scale; EEG, electroencephalogram; iEEG, intracranial electroencephalogram; WMS-IV, Wechsler memory scale-fourth edition; MADRS, Montgomery-Asberg depression rating scale; MRI, magnetic resonance imaging; fMRI, functional magnetic resonance imaging; DMN, default mode network.*

## Gamma Frequency Non-Invasive Brain Stimulation in Alzheimer’s Disease Patients

Sensory brain stimulation is currently the most commonly used technique to change brain waves activity in AD research. However, there are other approaches available which allow interference with brain activity and stimulate in desirable frequencies. Among these, transcranial alternating current stimulation (tACS), a non-invasive brain stimulation technique, has been proposed as an alternative technique to sensory stimulation and has the potential to result in stronger effects (Strüber and Herrmann, 2020). TACS delivers sinusoidal alternating electric currents over cortical areas of interest, at specific frequencies, entraining cortical oscillations at these frequencies. Hence, it has the potential to modulate cognitive functions that are related with the applied frequencies (for reviews please see Paulus, 2011; Herrmann et al., 2013).

Kehler et al. (2020) examined whether 40 Hz stimulation over the left dorsolateral prefrontal cortex, through tACS, could improve cognitive function in AD patients. Eleven patients with mild to moderate AD received two 30-min stimulation sessions per day, accompanied by cognitive exercises, for four consecutive weeks (active group) while six patients received cognitive exercises alone, for 4 weeks (control group). Memory improvements were observed in both groups, however, these improvements were maintained only to the active group at the follow up, at the 1-month post-treatment assessment.

In a recent study, a single session of 40 Hz tACS over Pz (an area overlying the medial parietal cortex and the precuneus) showed significant improvements on patients’ episodic memory performance and restoration of intracortical connectivity measures of cholinergic neurotransmission. The effect was evident in the AD patients who received the 40 Hz tACS, compared to sham exposure (Benussi et al., 2021). Even though the effects of 40 Hz tACS in AD underlying pathology were not considered, these results provide preliminary evidence that gamma frequency stimulation through non-invasive techniques may also have the potential to alleviate cognitive symptoms in AD patients. Increasing evidence suggests that tACS at gamma frequencies can effectively modulate a variety of cognitive functions in healthy and neuropsychiatric patients, demonstrating strong clinical potential for the inducement of gamma oscillations in AD (for a review please see Strüber and Herrmann, 2020).

Most recently, Liu et al. (2021) employed repetitive transcranial magnetic stimulation (TMS), a non-invasive brain stimulation technique that induces a pulsing electric current to the brain, which allows focused stimulation of the cerebral cortex, and demonstrated the clinical efficacy of 40 Hz in improving cognitive impairments in AD (for reviews on the use of TMS in AD see Nardone et al., 2014; Liao et al., 2015; Cheng et al., 2018; Taylor et al., 2019). The authors recruited 37 AD patients divided into active and sham TMS groups who received 12 sessions of 40 Hz TMS bilaterally at the angular gyrus, over the course of 4 weeks, and found significant cognitive improvement which lasted for up to 8 weeks in relation to sham stimulation. The authors also reported that 40 Hz TMS stimulation led to prevention of gray matter volume loss, and modulated gamma activity in the temporoparietal cortex as measured with power spectral density analysis of the resting-state electroencephalography (EEG) recordings, while at the same time increased local, long-range, and dynamic connectivity within the brain, enhancing information flow and integration. Importantly, the intervention was proven safe with no side effects. Accordingly, TMS might also have the potential to promote gamma rhythm synchronization, providing a novel non-pharmacological intervention for AD.

The non-invasive brain stimulation techniques provide the benefit of selecting specific brain areas for stimulation. Even though up to date no specific area has been identified as ideal to provide the most beneficial effects, it is suggested that several areas or pathways that are known to be negatively affected in AD, could provide potential targets. On that basis, by targeting these areas, crucial for normal cognitive function brain connections and pathways could be strengthened or rescued (e.g., stimulating the precuneus to alleviate abnormal functional connectivity in the default mode network) leading to cognitive improvements (Heath et al., 2018).

## Discussion and Future Directions

Gamma oscillations reflect a fundamental brain process, critical for healthy intra-brain communication and cognitive functions, especially memory, and, if disturbed, results in cognitive disfunction (Başar, 2013). Decreased gamma brain activity has been observed in both mice models of AD and human patients. Evidence, indicating aberrant gamma brain rhythms in mice, before the accumulation of amyloid plaques (Goutagny et al., 2013; Iaccarino et al., 2016) has drawn scientific interest toward the investigation of their role in AD pathology and their potential role in its treatment. Etter et al. (2019) showed that restoration of gamma oscillations in the hippocampus by optogenetic gamma stimulation of parvalbumin neurons is sufficient to rescue memory loss in mice without decrease of plaque load. In that respect, it is evident that gamma brain waves play a crucial role in memory function that appears to be independent from plaque accumulation.

Whether the observed disruption in gamma brain activity is responsible for the cognitive impairment in AD, or if it is just another outcome of the disease’s pathology remains to be answered. However, the entrainment of this brain activity and the consequent impact on the disease’s pathology has gained interest and has signified a new and promising era in AD research. Even though the investigations are still at their infancy, evidence suggests that restoration of gamma oscillations, through auditory and visual gamma stimulation, may have the potential to ameliorate AD pathology and improve cognition. The first evidence regarding the effectiveness of 40 Hz gamma entrainment through sensory stimulation in human patients (Chan et al., 2021; He et al., 2021), supports the notion regarding the essential role that gamma brain activity plays in AD pathology. In addition to sensory stimulation, scientists have investigated the effects of 40 Hz stimulation using other techniques that allow interference with brain activity and stimulate at desirable frequencies. For instance, non-invasive methods, such as transcranial alternating current stimulation and transcranial magnetic stimulation have been used generating positive results. On that basis, the new and promising research area of investigating the potential of gamma stimulation as a disease modifying therapeutic for AD, has emerged.

Cumulatively, current evidence from mice and human studies points toward investigating 40 Hz-based protocols as potential treatment for AD. Nevertheless, it should be noted that while the frequency-specific effect (i.e., 40 Hz) has been evident in mice studies (Iaccarino et al., 2016; Adaikkan et al., 2019; Martorell et al., 2019), the same remains to be determined in humans. Preliminary data from AD patient studies, do provide evidence about the potential therapeutic efficacy of 40 Hz entrainment. However, it remains unclear if these effects are specific to 40 Hz or can be seen using other gamma-based protocols. On that basis, it would be crucial in the future to compare 40 Hz stimulation with other gamma-range protocols, to determine whether the unique and profound effects of gamma stimulation are frequency-specific in humans.

A second interesting question that remains to be answered, is whether gamma stimulation targeting directly brain areas and connections that have been negatively affected by AD pathophysiology (e.g., the default mode network; Palop and Mucke, 2016), or cortical areas associated with the hippocampus, will be sufficient to restore gamma rhythms, AD pathology, and patients’ cognitive function. Finally, while several functions are attributed to gamma oscillations, such us their essential role in supporting fundamental circuit functions and cortical signal transmissions across the brain (Bosman et al., 2014), results from mice studies (Iaccarino et al., 2016; Adaikkan et al., 2019; Martorell et al., 2019) imply possible unappreciated mechanisms of action. In these studies, it is suggested that 40 Hz gamma stimulation can effectively attenuate mice pathology (i.e., Aβ and tau load; synapses and connectivity loss; cognitive function) by inducing profound glial responses and offering neuroprotection. It would be interesting therefore, to investigate the role of gamma oscillations in the recruitment of neuronal and glial responses, as well as in the modification of plasticity proteins. In contrast to mice studies, in humans, the observed improvements in patients’ cognitive functions have been attributed to the enhancement of gamma rhythm activity within the brain, which allows the information flow and integration (Liu et al., 2021). The effects of gamma stimulation on patients’ amyloid and tau burden as well as on microglial responses remain to be seen.

While gamma brain activity in AD patients is well-studied, the exact relationship between AD pathology and changes in gamma activity is yet inconclusive. Reduced gamma activity has been detected in AD patients (e.g., Stam et al., 2002; Başar et al., 2016) and has even been suggested as a potential biomarker for Mild Cognitive Impairment and AD (Murty et al., 2021). However, a number of studies have reported elevated gamma activity in AD patients (van Deursen et al., 2008; Başar et al., 2017). Although this contradictory observations may stem from various experimental parameters (Byron et al., 2021), a recent study demonstrated that changes in gamma brain activity might be dependent on the degree of amyloid burden (Gaubert et al., 2019). Hence, it is important for future studies to correlate AD pathology with changes in gamma oscillations (Byron et al., 2021) as well as to clarify the role that the degree of pathogenesis plays in gamma brain activity.

Further, although accumulated evidence indeed supports the idea that induction of gamma oscillations can modify AD pathology, several challenges and inconsistencies of this therapeutic approach remain. An important aspect for consideration is that cortical gamma rhythms can be induced *via* various mechanisms, such as the interneuron gamma (ING) and the pyramidal-interneuron gamma (PING; Tiesinga and Sejnowski, 2009; Buzsáki and Wang, 2012) and hence, outcomes and mechanisms of action may vary depending on the method used to increase gamma activity. For instance, Wilson et al. (2020) applied 40 Hz optogenetic stimulation in the basal forebrain parvalbumin neurons of mice models of AD and observed an increase of amyloid burden across several brain regions. As different methods of stimulation can lead to distinct molecular and cellular responses, thus implicating different mechanisms of action, it is of paramount importance to investigate which induction methods have beneficial or detrimental effects on patients’ pathology. Further research is needed to uncover these limitations in addition to well-designed clinical trials in which different ranges of gamma frequencies will be investigated.

Finally, considering that stimuli which can modulate gamma brain activity in humans are abundant in the environment (e.g., visual and auditory stimuli, such as human speech; Towle et al., 2008), an interesting question for future research is whether the effects of 40 Hz stimulation are specific to the stimulation protocols employed in the aforementioned studies or gamma brain activity can also be modulated by environmental sensory stimulation, such as everyday speech. Moreover, if indeed gamma sensory stimulation can alter brain gamma activity (e.g., Murty et al., 2018; Chan et al., 2021; Liu et al., 2021), such abundance of gamma sensory stimulation in the environment raises questions about how the brain protects interference by such stimuli.

In conclusion, the recent evidence on the potential therapeutic effects of gamma frequency stimulation in AD suggests that while induction of gamma oscillations is an important and promising approach for AD pathology alleviation, several issues remain to be addressed and much work remains to be done to establish whether this emerging field will lead to the development of novel AD therapeutic interventions.

## Author Contributions

AT and NK contributed to the conception and design of the study. AT wrote the first manuscript. NK contributed to the manuscript revision. Both authors approved the submitted version.

## Conflict of Interest

The authors declare that the research was conducted in the absence of any commercial or financial relationships that could be construed as a potential conflict of interest.

## Publisher’s Note

All claims expressed in this article are solely those of the authors and do not necessarily represent those of their affiliated organizations, or those of the publisher, the editors and the reviewers. Any product that may be evaluated in this article, or claim that may be made by its manufacturer, is not guaranteed or endorsed by the publisher.
